# Supplementary material for: The causal role of circulating inflammatory markers in osteoporosis: a bidirectional Mendelian randomized study
Source: Front Immunol. 2024 Jul 18;15:1412298. doi: 10.3389/fimmu.2024.1412298 (PMC11291241; doi:10.3389/fimmu.2024.1412298)
Supplement: Supplementary file 1 [file DataSheet_1.zip › supplementary material/CIM-OSTEOPOROSIS /GCST90274789.csv_leave_one_out.pdf]

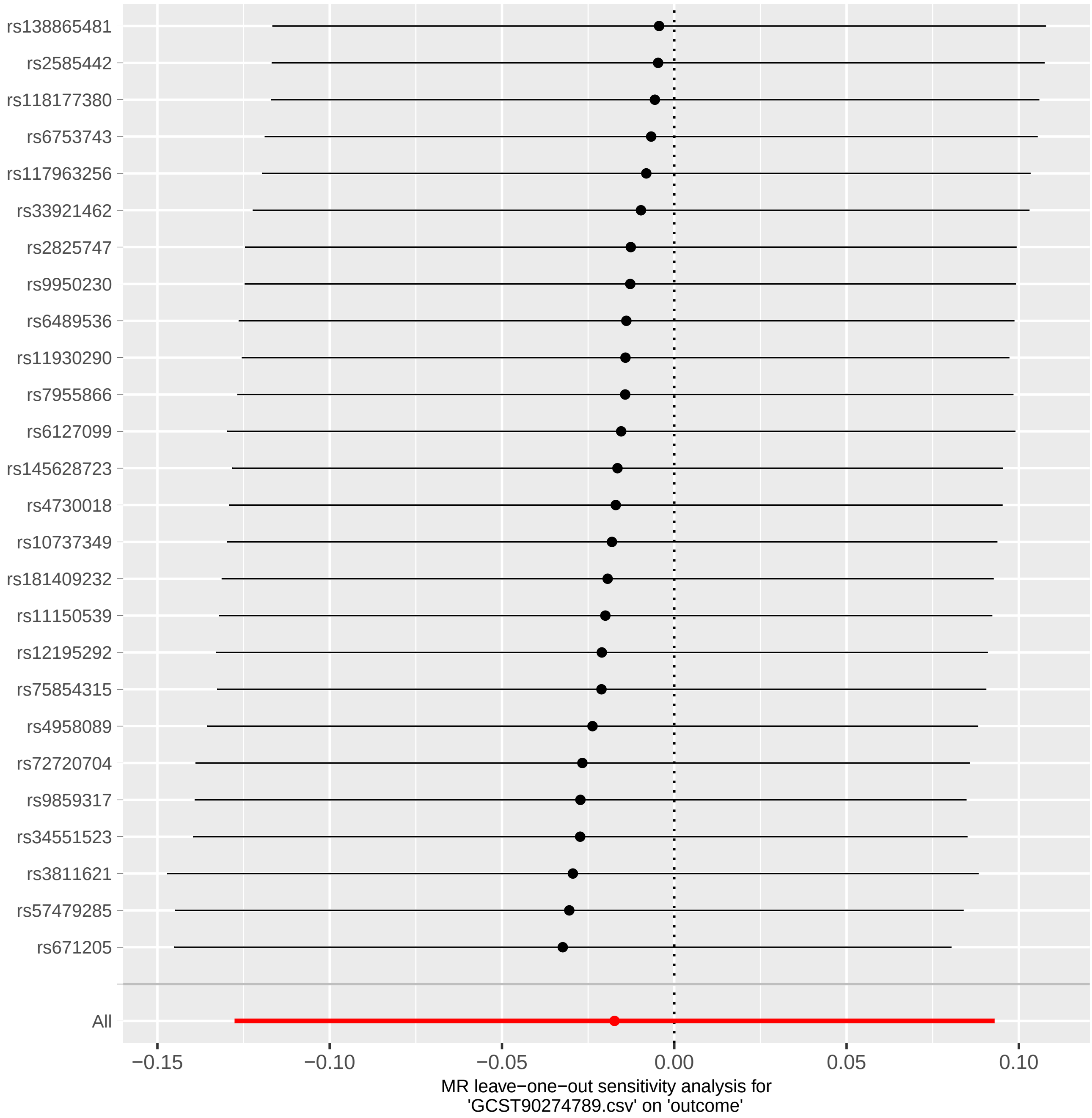
rs138865481
rs2585442
rs118177380
rs6753743
rs117963256
rs33921462
rs2825747
rs9950230
rs6489536
rs11930290
rs7955866
rs6127099
rs145628723
rs4730018
rs10737349
rs181409232
rs11150539
rs12195292
rs75854315
rs4958089
rs72720704
rs9859317
rs34551523
rs3811621
rs57479285
rs671205
All
−0.15
−0.10
−0.05
0.00
0.05
0.10
MR leave−one−out sensitivity analysis for
'GCST90274789.csv' on 'outcome'
